# Facile One-Pot Synthesis of Polydopamine Carbon Dots for Photothermal Therapy

**DOI:** 10.1186/s11671-018-2711-2

**Published:** 2018-09-17

**Authors:** Yuting Bai, Bai Zhang, Lu Chen, Zhenjie Lin, Xiuming Zhang, Dongtao Ge, Wei Shi, Yanan Sun

**Affiliations:** Key Laboratory of Biomedical Engineering of Fujian Province University/Research Center of Biomedical Engineering of Xiamen, Fujian Key Laboratory of Materials Genome, Department of Biomaterials, College of Materials, Xiamen University, Xiamen, 361005 China

**Keywords:** Carbon dots, Polydopamine, Photoluminescence, Photothermal therapy

## Abstract

Carbon dots (CDs) are a member of fluorescent carbon nanomaterials that are widely applied in bioimaging, photothermal therapy (PTT), and biosensors for its tunable fluorescence, photothermal conversion property, and excellent biocompatibility. Surface passivation and doping especially the doping of N atoms are critical factors to enhance the fluorescent intensity of CDs. Until now, a variety of nitrogen-rich molecules has been applied for the surface passivation of CDs such as L-Dopa, amino acids, and polyethylenimine (PEI). Herein, we report the synthesis of fluorescent polydopamine (PDA)-passivated carbon dots (CD-PDA) via one-pot microwave-assisted pyrolysis within 5 min, dramatically simplifying the reaction process compared with the hydrothermal treatment reported before. DLS, FT-IR, UV-Vis, and fluorescence spectroscopy were used to confirm the components of CD-PDA and to illuminate the mechanism of its tunable photoluminescence (PL). Due to the doping of N atoms by PDA, quantum yield (QY) of the CD-PDA was measured at 5%, which was nearly triple the original CDs without adding PDA. Yield of CD-PDA was about 1.5 times of the CDs on account of the enhancement of nucleation site for the carbon dot formation with the phenolic group provided by PDA. Meanwhile, photothermal conversion efficiency of the CD-PDA was determined to be 35% because of the excellent NIR light-thermal conversion property of PDA. Overall, we provided an extremely efficient approach to fabricate the fluorescent N-doped CD-PDA with stable photothermal conversion efficiency and excellent biocompatibility. More importantly, the passivation of PDA enabled the CD-PDA synthesized in our research compatible for further modification through Michael addition or Schiff base reaction.

## Background

As a member of low-dimensional carbon materials, the vast mixed SP^2^ and SP^3^ atoms as well as π-electrons in carbon dots (CDs) significantly augment defects and heteroatom of the photoactive systems, thus triggering the absorbed light energy into heat or the liberation of stimulated photon. The CDs have been widely applied in bioimaging, photothermal therapy (PTT), and biosensors for its tunable fluorescence, photothermal conversion property and excellent biocompatibility. The drug-loaded magnetofluorescent carbon quantum dots (MCQDs) synthesized via hydrothermal treatment and cross-linking reaction reported before have realized the combination of PTT and photodynamic therapy (PDT) through the fabrication of an efficient chemo-photo cancer therapy platform [1]. So far, extensive methods have been explored to enhance the fluorescent intensity of CDs since its first discovery during the purification of arc-discharged single-walled carbon nanotubes (SWCNTs) in 2004 [2], despite the synthesized routes to achieve certain extent of oxidation being general complicated. Top-down and bottom-up treatment are two common pathways to synthesize the CDs, including laser ablation [3, 4], oxidative acid treatment [5, 6], hydrothermal treatment [7, 8], microwave-assisted pyrolysis [9–11], electrochemical oxidation [12, 13], ultrasonic irradiation [14], and plasma treatment [15].

Researches show that the doping of N atoms is of great importance for the fluorescence enhancement of the CDs [16–18]. Liu et al. used polyethylenimine (PEI) providing the N atoms to fabricate PEI-functionalized CDs by one-step microwave-assisted (700 W) pyrolysis of glycerol and branched PEI; quantum yield (QY) of the system was measured up to 15.3%, and it was applied for cell imaging and gene delivery [19]. Zhou et al. reported the development of phosphor and nitrogen co-doped carbon dots (N-P-doped CDs) for bioimaging via the hydrothermal treatment of nucleotide adenosine-5′-triphosphate (ATP) at 180 °C for 10 h. Typically, ATP was the sole material source for the doping of both N and P atoms to enhance defects on the surface in the system, thus leading to augmentation of QY of the N-P-doped CDs (calculated at 9.8%) [20]. In addition, it was reported that phenolic compounds could serve as a catalytic seed for the growth of carbon dots. Lee et al. found that carbon dots were dramatically increased with adding a trace amount of ferulic acid [21].

Polydopamine (PDA) is a kind of melanin-like polymer derived from the polymerization of dopamine (DA) monomer, which has been widely applied for surface modification of various materials since it was firstly studied as an adhesive surface modification agent [22]. As we all know, vast amounts of N-rich and phenolic hydroxyl functional groups such as catecholamine in PDA make it a potentially excellent passivant and catalyst for the CDs.

Inspired by this, we report a facile efficient one-pot microwave-assisted pyrolysis pathway for the synthesis of PDA-functionalized CDs over 5 min. Dynamic light scattering (DLS), Fourier transform infrared spectroscopy (FT-IR), transmission electron microscopy (TEM), ultraviolet and visible spectroscopy (UV-Vis), and fluorescence spectroscopy were used to define the components of polydopamine carbon dots (CD-PDA) and its tunable photoluminescence (PL). Relative cell viabilities of HeLa cells treated by CD-PDA with and without NIR irradiation were measured by standard 3-(4, 5-dimethylthiazol-2-yl)-2, 5-diphenyltetrazolium bromide (MTT) assay.

## Results and Discussion

### Synthesis and Characterization of CD-PDA

**Scheme 1:**
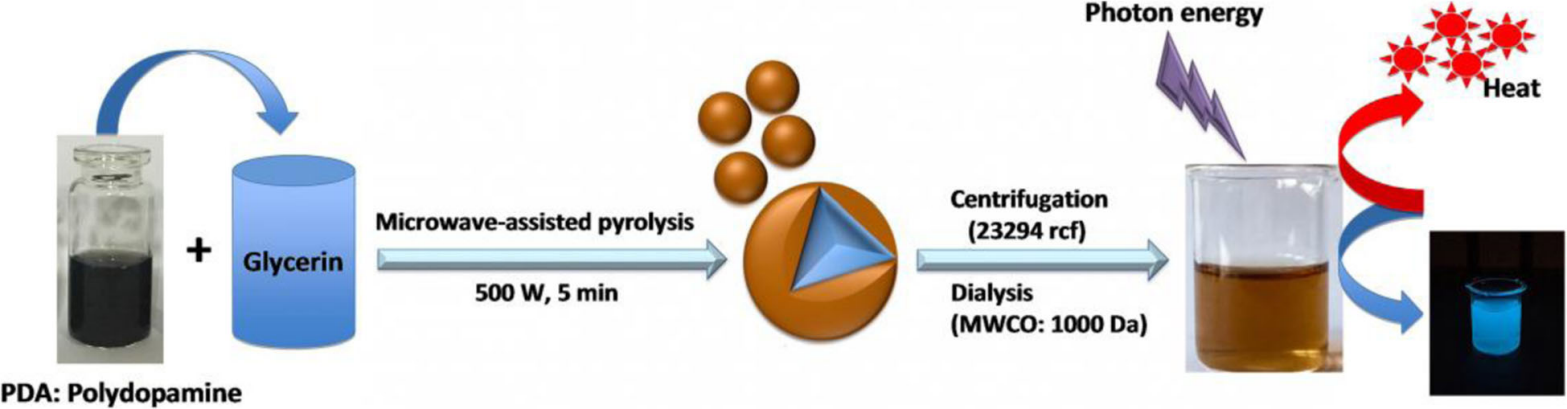
Schematic illustration of the synthesis process of CD-PDA

In this research, we synthesized the polydopamine (PDA)-functionalized carbon dots (CD-PDA) via one-pot microwave-assisted pyrolysis of glycerin and PDA. The carbon dots (CDs) fabricated via the same microwave-assisted pyrolysis approach without adding PDA was set as a control group. Schematic illustration of the synthesis process of CD-PDA was described conceptually in Scheme 1. Yield of the CD-PDA was nearly 1.5 times that of the CDs, due to the enhancement of nucleation site for the carbon dot formation with the phenolic group provided by PDA [21].

**Fig. 1:**
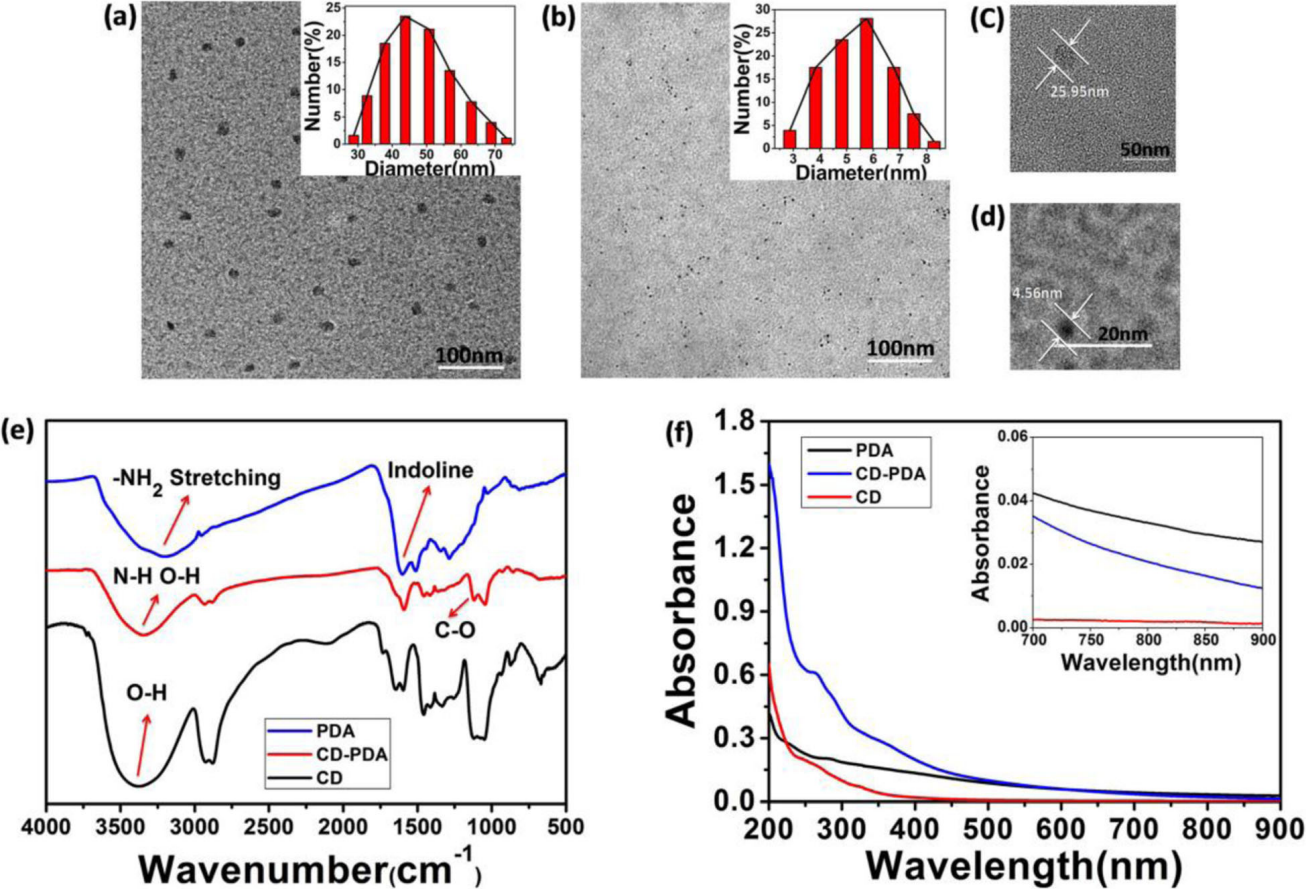
Morphology, FT-IR spectra, and UV-Vis spectra of CD-PDA and CDs. **a** TEM image of CD-PDA (scale bar 100 nm, inset: size distribution determined by DLS). **b** TEM image of CDs (scale bar 100 nm, inset: size distribution determined by DLS). **c** Zoomed-in image of a single CD-PDA (scale bar 50 nm). **d** Zoomed-in image of a single CD (scale bar 20 nm). **e** FT-IR spectra of CD-PDA, CDs, and PDA. **f** UV-Vis spectra of CD-PDA, CDs, and PDA (inset: the absorbance from 600 to 900 nm)

Dynamic light scattering (DLS) profiles revealed the particle size and zeta potential of CD-PDA. The hydrodynamic particle size of CD-PDA was 51.5 ± 19.5 nm (inset of Fig. 1a) and the zeta potential was determined to be − 27.5 ± 0.4 mV, indicating negatively charged groups on the surface of the nanodots, which further demonstrated the surface modification by PDA. The hydrodynamic particle size of CDs dispersed in DI water was 5.5 ± 2.5 nm (inset of Fig. 1b). Transmission electron microscopy (TEM) images characterized the monodisperse spherical and uniform size-distributed nanoparticles (Fig. 1a, b); the diameter of CD-PDA was ~ 25 nm (Fig. 1c) and that of CDs (Fig. 1d) was measured at ~ 5 nm. After the surface modification by PDA, growth of the diameter of CD-PDA was about 20 nm compared with that of CDs.

The passivation of PDA on the carbon dots was recorded by FT-IR spectra. Here, PDA was synthesized via the polymerization of 20 mg DA hydrochloride in 10 mL Tris buffer (pH 8.5, 10 mM) at room temperature for 12 h and then it was centrifuged at 23,294 rcf. As the spectrum information of characteristic peaks of CD-PDA, CDs, and PDA observed in Fig. 1e, the peak of 3400 cm^−1^ and 1600 cm^−1^ suggested the catechol –OH groups and aromatic rings of PDA, which also existed in the CD-PDA [23, 24]. New peaks appearing at 1642 cm^−1^, 1588 cm^−1^, and 1640 cm^−1^ referred to C=O, N–H, and C–N while the peak at 3400 cm^−1^ indicated the existence of –OH and N–H in the system, which further illustrated the surface modification of PDA on the CDs. The N–H, C=O, and C–N emerging in the carbon dots during the microwave-assisted oxidation demonstrated the mechanisms of the surface passivation for the nanodots: during the 5-min microwave-assisted oxidation were the polymerization of dopamine and the dehydration of system to form the core of the nanodots, after which was the growth of the carbon dots.

The UV-Vis absorption spectra of CD-PDA, CDs, and PDA with the same concentration are shown in Fig. 1f (concentration of the sample inset Fig. 1f, 12.5 μg/mL). As the surface passivant of the system, PDA exhibited broad-spectrum absorption from 200 to 900 nm especially in the near-infrared region, which was essential for the excellent photothermal conversion property of CD-PDA. The absorption at 220 nm and 280 nm represented the electron transition among the strong π-stacking of phenyl ring as a conjugated system, verifying the modification of PDA. Particularly, the obvious reduction of absorption at 280 nm indicated the spatial barrier between the strong π-stacking interactions in the conjugated system after the passivation of PDA [25], while the UV-Vis absorption spectra of CD-PDA show the characteristic peaks around 274 nm and 370 nm, and that of CDs was measured at 260 nm and 330 nm. The bathochromic shift from 330 to 370 nm was accounted for the introduction of amidogen from the PDA, which was also characterized for the chelation of the glycerin and PDA [26, 27]. The inset is the absorbance of CD-PDA, CDs, and PDA from the wavelength 600 to 900 nm.

### Photoluminescence of CD-PDA

**Fig. 2:**
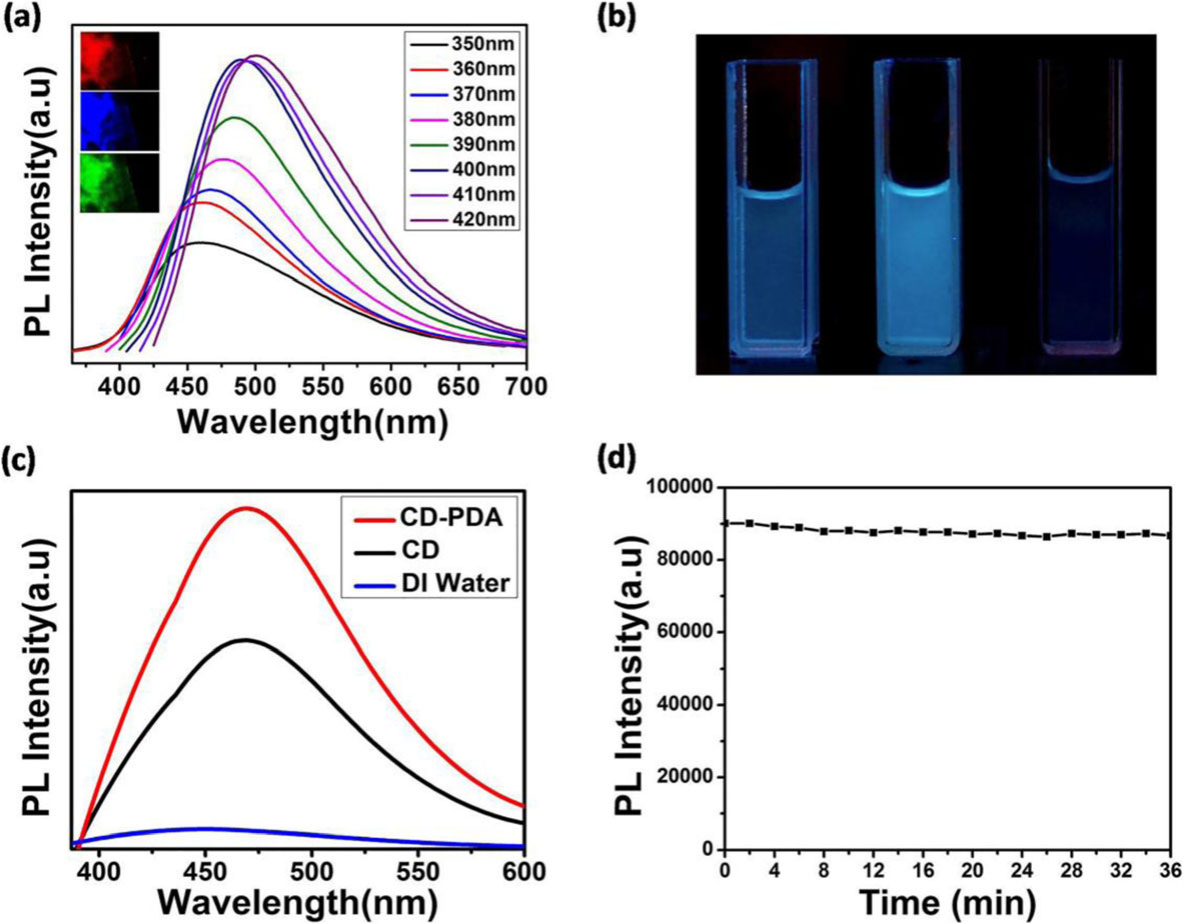
Optical properties of CD-PDA and CDs. **a** Photoluminescence spectra of CD-PDA (excitation wavelengths range from 350 to 420 nm with 10 increments, inset: fluorescent microscopy images of CD-PDA). **b** From left to right: CDs, CD-PDA, and DI water under the UV light irradiation (365 nm). **c** Photoluminescence spectra of CD-PDA, CDs, and DI water. **d** Stability curve of the fluorescent intensity of CD-PDA

As reported before, surface modification of the carbon dots can affect its photon conversion process to a great extent, thus leading to tremendous diversity in the fluorescence spectra [28, 29]. In our research, the emission peak of CD-PDA red-shifted from 450 to 500 nm with the excitation wavelength changed from 350 to 420 nm (Fig. 2a). Accordingly, we observed red, blue, and green fluorescent microscopy images by dipping drops on a glass slide, further clarifying the tremendous diversity of fluorescence for CD-PDA (inset of Fig. 2a). Moreover, we detected the macroscopic images of the CD-PDA, CDs, and DI water under the UV light illumination (365 nm), confirming that the fluorescent intensity of CD-PDA was much stronger than that of CDs (Fig. 2b). Figure 2c further illustrates the enhancement of fluorescent intensity after the surface modification of PDA; the quantum yield (QY) of CD-PDA was nearly triple that of the CDs (quinine sulfate was selected as the standard sample [19]), verifying the effect of doping N atoms from PDA. We tested the stability of fluorescent intensity of CD-PDA; it did not show a clear change under the 2100-s irradiation (365 nm), hence exhibiting stable photoluminescence property (Fig. 2d).

**Fig. 3:**
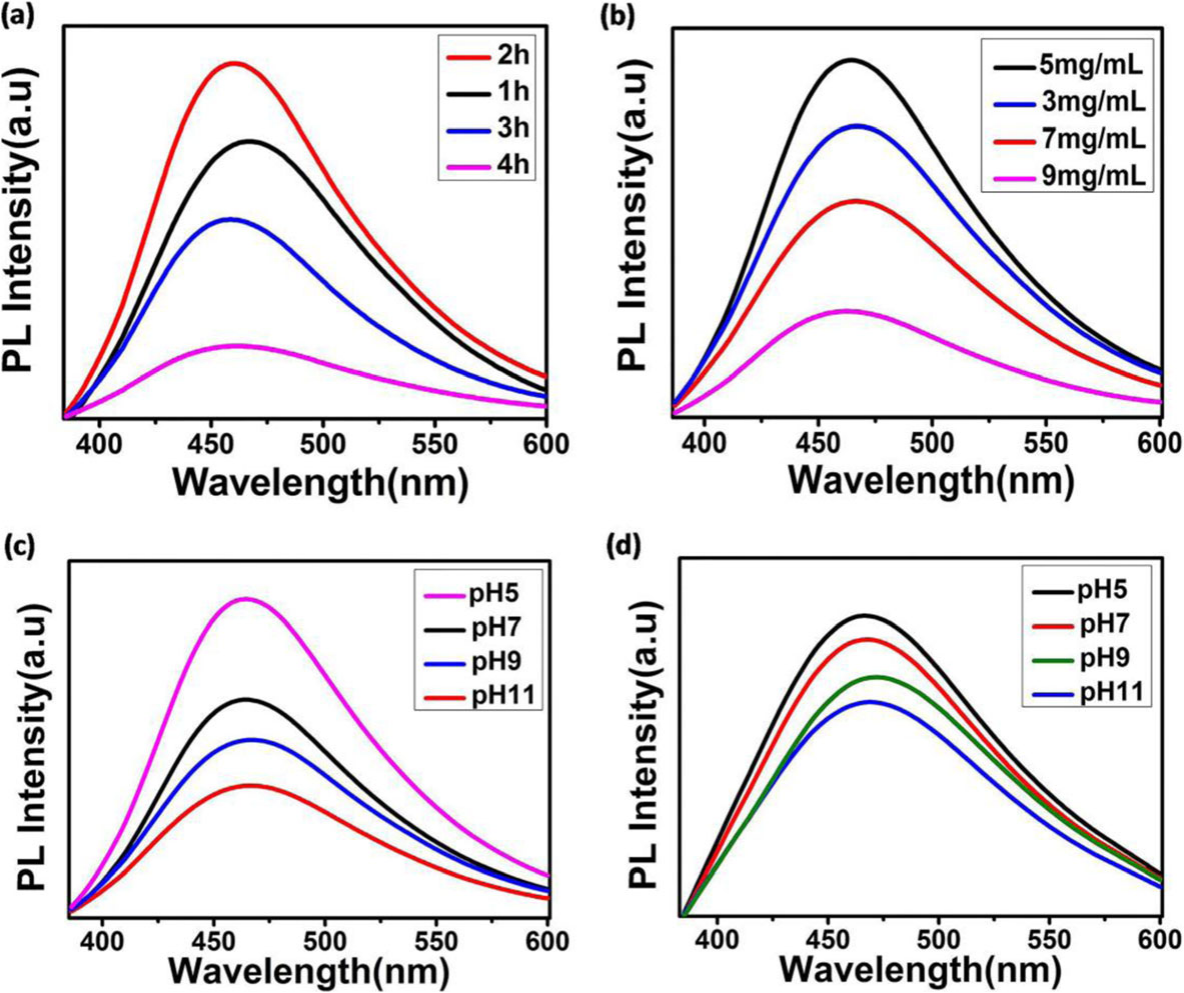
Photoluminescence spectra of CD-PDA. **a** Fluorescent intensity of CD-PDA with various duration of dopamine polymerization in Tris buffer. **b** Fluorescent intensity of CD-PDA with various original dopamine concentrations. **c** Fluorescent intensity of CD-PDA with different pH before the microwave-assisted pyrolysis. **d** Fluorescent intensity of CD-PDA with different pH after the microwave-assisted pyrolysis

To further study the influence of PDA for the enhancement of fluorescent intensity of CD-PDA, we firstly measured the fluorescent intensity against duration of the polymerization of dopamine in Tris buffer. The gradient of photoluminescence in Fig. 3a illustrated that CD-PDA with dopamine polymerize in Tris buffer for 2 h exhibited the highest fluorescent intensity, indicating the influence of the extent of dopamine pre-polymerization. We further explored the fluorescent intensity of CD-PDA with various original PDA concentrations in Tris buffer (3, 5, 7, and 9 mg/mL). As the original DA concentrations vary from 3 to 9 mg/mL, the fluorescence of CD-PDA presented the tendency of firstly increasing and then decreasing (Fig. 3b).

Furthermore, we investigated the fluorescent intensity of CD-PDA with various initial pH; the fluorescent intensity decreased as the pH of the Tris buffer increased from 5 to 11 (Fig. 3c). Figure 3d exhibited the influence of pH after the microwave-assisted oxidation. The pH of the system after microwave-assisted oxidation was mediated from 5 to 11, and we compared the fluorescent intensity of CD-PDA; acid medium (pH 5.0) lead to stronger fluorescence, which also indicated stronger fluorescence of CD-PDA in the acidic tumor microenvironment.

### Photothermal Performance and Cytotoxicities of CD-PDA

#### Measurement of Photothermal Efficacy

**Fig. 4:**
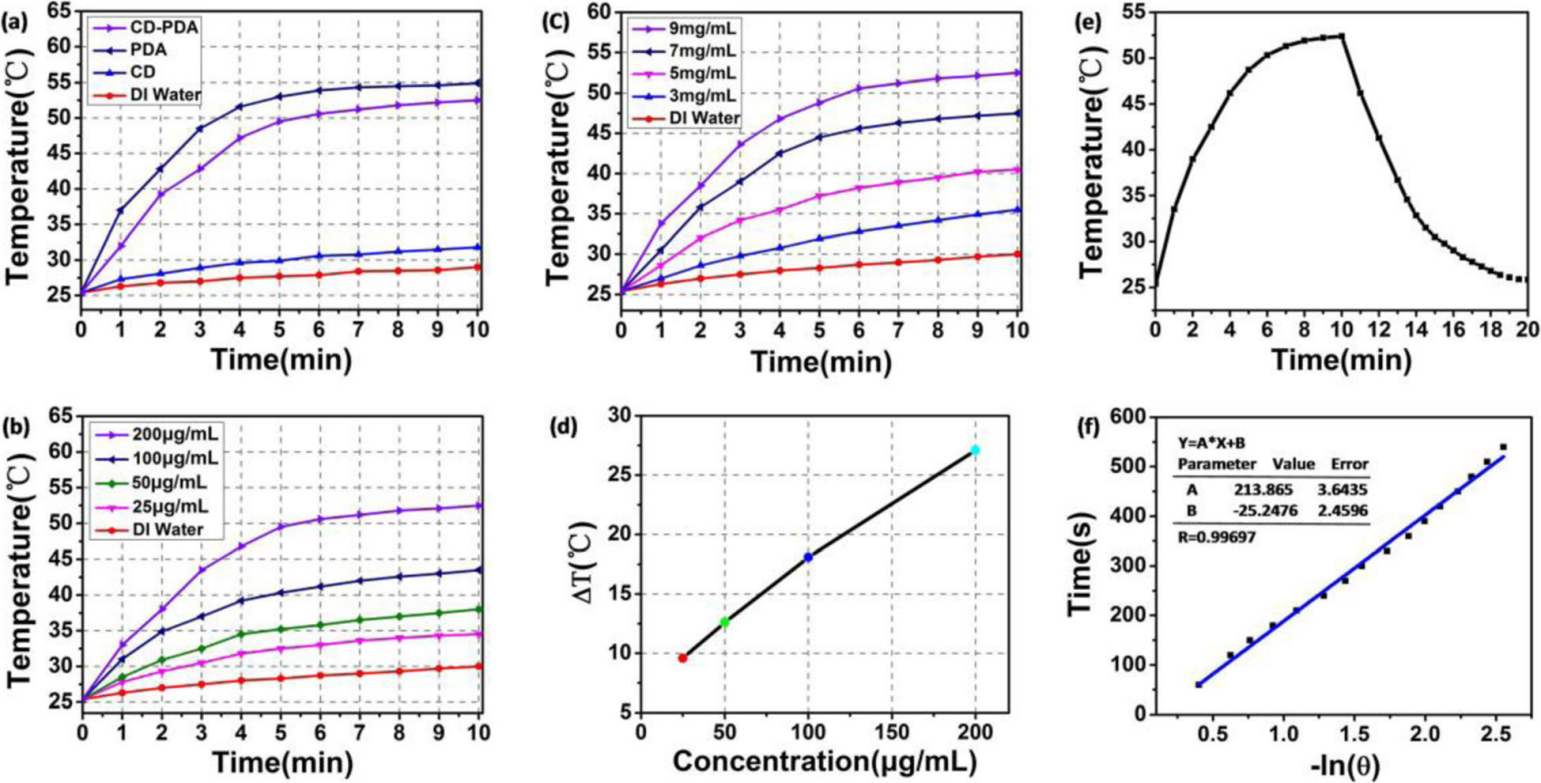
Photothermal conversion properties of CD-PDA and CDs. **a** Photothermal heating curves of CD-PDA, CDs, PDA, and DI water under a power density of 2 W/cm^2^ NIR laser irradiation for 10 min. **b** Photothermal heating curves of CD-PDA at various concentrations during 10 min. **c** Photothermal heating curves of CD-PDA (200 μg/mL) with various original DA concentrations in Tris buffer. **d** Temperature increment of CD-PDA at various concentrations. **e** Cooling curve of CD-PDA (under a power density of 2 W/cm^2^ NIR irradiation in the first 10 min and naturally cooling to the room temperature). **f** Leaner time data versus − lnθ calculated according to the cooling curve of CD-PDA

To demarcate the analysis of the photothermal conversion efficiency of CD-PDA and CDs, we quantitatively evaluated the temperature increment against time under irradiation; PDA was selected as an additional control group. Under 10 min irradiation (808 nm, 2 W/cm^2^), the temperature increment of CD-PDA was 27 °C while that of PDA was about 30 °C at 200 μg/mL. Meanwhile, temperature increment of CDs (200 μg/mL) under irradiation during the 10 min was about 7.5 °C and that of DI water was no more than 5 °C (Fig. 4a). Furthermore, we measured the elevation of temperature of the CD-PDA at various concentrations as a function of time under a power density of 2 W/cm^2^ NIR laser irradiation during 10 min. Overall, the enhancement of temperature grew with the increase of concentration of the CD-PDA, and the temperature increased faster as the concentration of CD-PDA increased from 25 to 200 μg/mL (Fig. 4b). Whereupon, we draw the curve of the temperature increment against various concentrations of CD-PDA, among which the temperature increment of CD-PDA at 200 μg/mL, 100 μg/mL, 50 μg/mL, and 25 μg/mL was about 27 °C, 18 °C, 13 °C, and 10 °C, respectively (Fig. 4d). Typically, in order to study the influence on the photothermal conversion efficiency of CD-PDA against various initial concentrations of DA in Tris buffer, we measured the temperature change of CD-PDA (200 μg/mL) with various original concentrations of DA in Tris buffer (Fig. 4c). The temperature increased as the concentration of DA improved from 3 to 9 mg/mL. The increase of temperature was 27 °C when the original concentration of DA was 9 mg/mL while the temperature increment was only 10 °C when the original concentration of DA was 3 mg/mL. The naturally cooling curve of CD-PDA is presented in Fig. 4e (200 μg/mL, 808 nm, 2 W/cm^2^, 20 min), and the leaner data of − lnθ calculated from the cooling period is observed in Fig. 4f. The photothermal conversion efficiency of CD-PDA was measured at 35%, higher than that of the Au nanorods reported before (literature value, 22% [30]).

#### In Vitro Cell Viability

**Fig. 5:**
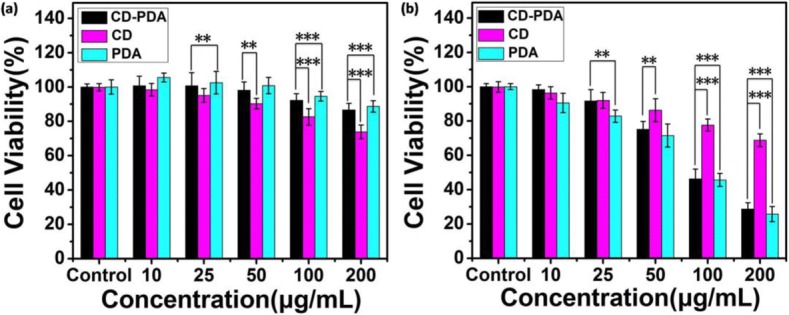
In vitro cytotoxicity against HeLa cells. **a** In vitro cell viability of HeLa cells incubated with CD-PDA, CDs, and PDA at various concentrations for 24 h. **b** In vitro cell viability of HeLa cells incubated with CD-PDA, CDs, and PDA at various concentrations under irradiation (808 nm, 2 W/cm^2^, 5 min; mean ± SD, *n* = 6). *p < 0.05, **p < 0.01, ***p < 0.001

The cytotoxicities of CD-PDA, CDs, and PDA were analyzed by a standard MTT assay. To evaluate the differences of cell viability among CD-PDA, CDs, and PDA, HeLa cells were incubated with these nanoparticles at the same concentration in each group. The MTT results (Fig. 5a) revealed that the cell viability of HeLa cells exhibited a dose-dependent relationship with the CD-PDA, CDs, and PDA. It was reported that the quinone-rich PDA-modified surface was energetic in the activity of cell proliferation [31]. It is notable in our study that the CD-PDA could obviously promote the cell viability of HeLa cells even at the concentration of 50 μg/mL due to the surface modification by PDA and the cell viability was not dramatically inhibited at 100 μg/mL, which basically shared the same tendency with the results of PDA, whereas viability of HeLa cells that were incubated with the CDs reduced to 80% and 70% at 100 μg/mL and 200 μg/mL, respectively.

The standard MTT assay was further assessed on HeLa cells to determine the photothermal killing efficiency of CD-PDA, CDs, and PDA, HeLa cells were incubated with these nanoparticles at the same concentration in each group. While under irradiation (808 nm, 2 W/cm^2^, 5 min), the MTT assay (Fig. 5b) showed that the photothermal killing efficacy of CD-PDA, CDs, and PDA was enhanced as a function of their concentration. Overall, the cell viability of HeLa cells incubated with CD-PDA reduced to 30% at 200 μg/mL, manifesting the photothermal killing efficacy of the system. Meanwhile, it is noteworthy that the difference of cell viability among CD-PDA and PDA decreased progressively as their concentration increased from 25 to 200 μg/mL thanks to the obvious temperature increment of CD-PDA under NIR irradiation along with the improvement of its concentration (Fig. 4b, 4d). Moreover, cell viability of HeLa cells incubated with CDs under NIR irradiation was 68% at 200 μg/mL, which was not a significant variation compared with that at the same concentration without NIR laser due to its weak light absorption at the near-infrared region (Fig. 1e).

## Conclusions

In this work, we report the synthesis of fluorescent polydopamine (PDA)-passivated carbon dots (CD-PDA) via one-pot microwave-assisted pyrolysis within 5 min, dramatically simplifying the reaction process, promoting its fluorescent intensity due to the doping of N atoms from PDA, and improving its yield because of the enhancement of nucleation site for carbon dot formation with the phenolic group provided by PDA. After the passivation of PDA, yield of CD-PDA was nearly 1.5 times of the CDs; quantum yield of CD-PDA was ~ 5%, tripled that of the original CDs. Photothermal conversion efficiency of the system was measured at 35%, higher than that of Au nanorods reported before (22%). During in vitro test, the CD-PDA exhibited excellent biocompatibility and the performance of PTT; it could even promote the cell viability of HeLa cells with the concentration arriving at 50 μg/mL. While under irradiation, the cell viability of HeLa cells reduced to 30%. More importantly, the passivation of PDA enabled the system compatible for further modification through Michael addition or Schiff base reaction.

## Methods/Experimental

### Materials

All chemical reagents were of analytical grade and used without further purification unless otherwise stated. Dopamine hydrochloride (DA) was purchased from Sigma-Aldrich (USA); quinine sulfate (98%, fit for fluorescence) was obtained from Fluka (USA); and glycerin (> 99%), Tris, dimethyl sulfoxide (DMSO, > 99.8%), and dialysis membranes (MWCO 1000 Da) were supplied by Sangon Biotech (Shanghai, China). 3-(4, 5-Dimethylthiazol-2-yl)-2, 5-diphenyltetrazolium bromide (MTT), trypsin, and penicillin-streptomycin solution were obtained from Beyotime Biotechnology (Shanghai, China). Dulbecco’s modification Eagle medium (DMEM) was attained from Hyclone (USA). Fetal bovine serum (FBS) was purchased from Biological Industries (Israel). HeLa cells were provided by American Type Culture Collection (ATCC).

### Instrumentation and Characterization

Elementary composition was confirmed by Fourier transform infrared spectroscopy conducted on the Nicolet 380 spectrometer (FT-IR, Thermo Nicollet, Instruments, Ltd., America). UV-Vis spectra were characterized by Perkin Elmer Lambda 750 UV-vis near-infrared spectrophotometer (UV-vis-NIR, Perkin-Elmer, Norwalk, CT). The photoluminescence (PL) spectra were measured by Infinite 200PRO Fluorometer (Tecan, Instruments, Ltd., Switzerland). Diameter distribution and zeta potential were performed by Mastersizer2000 (DLS, Nano-ZS, Malvern, Instruments, Ltd., UK). Morphology and diameter were represented by transmission electron microscopy (TEM, Tecnai G, Spirit, FEI, Hong Kong). Domestic microwave oven was served as the microwave source (500 W) and reaction still (Galanz, Instruments, Ltd., China).

### Preparation of CD-PDA and CDs

Firstly, 50 mg dopamine hydrochloride was completely dissolved in 10 mL Tris buffer (10 mM, pH 8.5) and self-polymerized at room temperature for 2 h under magnetic stirring. Then, the CD-PDA was synthesized by directly mixing 6 mL pre-polymerized PDA solution above and 20 mL glycerin (> 99%) before 5 min microwave-assisted (500 W) oxidation and the follow-up purification step. While the CDs were prepared by 5 min microwave-assisted (500 W) oxidation of 20 mL glycerin, it was set as a control group. Thereafter, both CD-PDA and CDs were purified via dialysis against DI water for 48 h (MWCO 1000 Da) and finally collected by centrifugation (23,294 rcf, 10 min) and lyophilization.

### Measurement of Fluorescent Quantum Yields

Quantum yield (QY) of CD-PDA was measured via the colorimetric method reported before [19], quinine sulfate (in 0.1 M H_2_SO_4_) was selected as the standard sample (literature QY 54%), and the photoluminescence (PL) emission was measured by Infinite 200PRO Fluorometer. Overall, the specific value for fluorescent intensity of CD-PDA and quinine represented the QY of CD-PDA (excitation wavelength 350 nm) on condition that they shared the same optical density (OD) value less than 0.02 (wavelength 350 nm). The integrated fluorescent intensity was the area beneath the PL curve with the wavelength ranging from 380 to 700 nm. Basically, quinine sulfate dissolved in 0.1 M H_2_SO_4_ was served as the standard sample (OD value 0.02, wavelength 350 nm); CD-PDA was dispersed in DI water, and we mediated its OD value to 0.02 in order to exclude the influence of light absorption. Then, we measured the fluorescent intensity of CD-PDA and quinine to calculate area of the PL curves. The CDs were set as a control group. Absolute value of the QY was calculated according to the formula:

$$ {F}_X={F}_{ST}\left(\frac{{\mathrm{Grad}}_X}{{\mathrm{Grad}}_{ST}}\right)\left(\frac{R_X^2}{R_{ST}^2}\right) $$

Thereinto, *F* is the QY, Grad is gradient of the PL curve, ST and X represent the standard and test group, respectively, and *R* is refractive index of the solvent.

### Measurement of Photothermal Performance

CD-PDA, CDs, and PDA were all dispersed in DI water and their concentrations were all mediated at 200 μg/mL. Then, we added 1 mL solution above into the standard quartz cell, respectively, and set the laser diode source (STL 808CFS-10W, China) above the liquid level about 1 cm in order to completely cover the solution. We measured the temperature changes of CD-PDA and CDs every minute under a power density of 2 W/cm^2^ NIR laser irradiation; both PDA and DI water were set as control groups. Then, we ended the irradiation and recorded the temperature changes as the CD-PDA naturally cools to the room temperature to draw the cooling curve. Photothermal conversion efficiency of CD-PDA was calculated according to the formula reported before [30].

### Cell Culture

HeLa cells were cultured in Dulbecco’s modification Eagle medium (DMEM, HyClone) containing high glucose with 10% fetal bovine serum (FBS), penicillin (100 U/mL), and streptomycin (100 μg/mL) at the temperature of 37 °C and 5% CO_2_ humidified atmosphere. We changed the culture medium once a day.

### Cell Viability Assay

The cytotoxicity of CD-PDA was measured via standard MTT assay. HeLa cells were seeded in 96-well plates with a density of 2 × 10^4^ cells per well and cultured for 24 h at 37 °C, 5% CO_2_ humidified atmosphere. Then, we cleaned the HeLa cells three times with fresh PBS, after which the CD-PDA dispersed in DMEM with various weight ratios (10, 25, 50, and 100 μg/mL) was added into each well. After, it was incubated for another 24 h at 37 °C, 5% CO_2_ humidified atmosphere. The culture medium was replaced by 200 μL DMEM containing 20 μL MTT (5 mg/mL in PBS) and incubated for another 4 h at 37 °C, 5% CO_2_ humidified atmosphere. Finally, we thoroughly removed the medium and added 200 μL DMSO into each well, shaking for another 15 min. The absorbance of each well was measured at 490 nm. Non-treated HeLa cells (cultured in DMEM) were set as a control group. The relative cell viabilities of HeLa cells were calculated according to the formula Abssample/Abscontrol × 100%. Thereinto, the Abssample is absorbance of HeLa cells treated by CD-PDA while the Abscontrol represents absorbance of non-treated HeLa cells.

## Abbreviations

CD-PDA: Polydopamine carbon dots
CDs: Carbon dots
DA: Dopamine
DLS: Dynamic light scattering
DMEM: Dulbecco’s modification Eagle medium
DMSO: Dimethyl sulfoxide
FBS: Fetal bovine serum
FT-IR: Fourier transform infrared spectroscopy
MTT: 3-(4, 5-Dimethylthiazol-2-yl)-2, 5-diphenyltetrazolium bromide
NIR: Near-infrared region
OD: Optical density
PDA: Polydopamine
PEI: Polyethylenimine
PL: Photoluminescence
PTT: Photothermal therapy
QY: Quantum yield
SWCNTs: Single-walled carbon nanotubes
TEM: Transmission electron microscopy
UV-Vis: Ultraviolet and visible spectroscopy
